# Supplementary material for: Heat adaptation of phage T7 under an extended genetic code
Source: Virus Evol. 2021 Dec 1;7(2):veab100. doi: 10.1093/ve/veab100 (PMC8923235; doi:10.1093/ve/veab100)
Supplement: veab100_Supp [file veab100_supp.zip › Sequencing_Analysis.zip!/Sequencing_Analysis/A3/evidence/JC_SIDE_2_73.html]

BRESEQ :: Evidence


| New junction evidence | | | | | | | | | | | |
| --- | --- | --- | --- | --- | --- | --- | --- | --- | --- | --- | --- |
|  | | seq id | position | reads (cov) | reads (cov) | score | skew | freq | annotation | gene | product |
| \* | ? | NC\_001604.1 | = 13586 | 0 (0.000) | 152 (0.090) | 52/592 | 21.8 | 51.1% | intergenic (–/–) | *–/–* | –/– |
|  | ? | NC\_001604.1 | 14356 = | 291 (0.170) |  |  |  |  | intergenic (–/–) | *–/–* | –/– |
| Rejected: Coverage evenness skew score above cutoff. | | | | | | | | | | | |

Only 100 of 1220 total aligned reads displayed.  

`GACAGACTGGCTGGAAGAAACCAGAAGTTACCAAGCTGAGTCATAAGGTGTTCGATAAGGACACTATGACCCACATCAAGGCTGGTGATTGGGTTAAGGTTGACATGGGAGTTGTTGGTGGATACGGCTACGTCCGCTCAGTTAGTGGCAAATATGCACAAGTGTCATACATCACAGGTGTTACTCCACGCGGTGCAATCGTTGCCGATAAGACCAACATGATTCACACAGGTTTCTTGACAGTTGTTTCATATGAAGAGATTGTTAAGTCACGATAATC‑AATAGGAGAAATCAATATGATCGTTTCTGACATCGAAGCTAACGCCCTCTTAGAGAGCGTCACTAAGTTCCAC‑TGCGGGGTTATCTACGACTACTCCACCGCTGAGTACGTAAGCTACCGTCCGAGTGACTTCGGTGCGTATCTGGATGCGCTGGAAGCCGAGGTTGCACGAGGCGGTCTTATTGTGTTCCACAACGGTCACAAGTATGACGTTCCTGCATTGACCAAACTGGCAAAGTTGCAATTGAACCGAGAGTTCCACCTTCCTCGTGAGAACTGTATTGACACCCTTGTGTTGTCACGTTTGATTCATTCCAACC  >  NC_001604.1/14057‑14656`  

                                                                                                                                                                                                                                                                                                            `|`                                                                                                                                                                                                                                                                                                               
`ggaaaggtgtataagggacagattattgcccttgagcgtaagcagcaaggcgatatgactaaccctttcctcgttagtattcgcaagtgccgctttactggtgatactggtatcgctggctacatggaatacaacaaggaaaccagatggcttgaaccatcaagttactcagtggaagaagagtcacactcagagtcaacagactggtcaaacgacactgacttctgacaggattcttgaCATTTGTTTCATATGAAGAGATTGTTAAGTCACGATAATC‑AATAGGAGAAATCAATATGAt                                                                                                                                                                                                                                                                                                              <  1:118477‑M2/61‑1 (MQ=255)`  
     `ccaactatctgatactattattgcccttgagcgtaatcagcaaggcgatatgcctaaccttgtcctcgttcgtattctcaagtgccgctttactggtgatactggtatcgctggctacatggaatacaacaaggaaaccggatggcttgaaccatcaagttactcaggggaagaagagtcacactcagagtcaacagactggtccaacgacactgacttctgacaggattcttgaCAGTTGTTTCATATGAAGAGATTGTTAAGTCACGATAATC‑AATAGGAGAAATCAATATGATCGttt                                                                                                                                                                                                                                                                                                         >  1:224725‑M2/236‑301 (MQ=255)`  
        `attatctgagactattcttgcccttgagcataatcagcaaggcgatatgccttaacttgtcctcgttcgtattctacagtgccgctttacttgtgattctggtatcgctggctacatggaatacaacaaggaaaccggatggcttgaaccatcaaggtactcaggagaagaagagtcacactcagagtcaacagactggtcaaacgacaatgacttctgacaggattcttgaCAGTTGTTTCATATGAAGAGATTGTTAAGTCACGATAATC‑AATAGGAGAAATCAATATGATCGTTTCTg                                                                                                                                                                                                                                                                                                      <  1:334477‑M2/69‑1 (MQ=255)`  
         `ctatctgatactattattgcccttgagcgtaatcagcaaggcgatatgcctaaccttgtcctcgttcgtattctcacgtgccgctttactggtgatactggtatcgctggctacatgtaatacaacaaggaaaccggatggctttaaccatcaagttactcaggggaagaagagtcacactcatagtcaacagactggtccaacgacactgacttctgacaggattcttgaCAGTTGTTTCATATGAAGAGATTGTTAAGTCACGATAATC‑AATAGGAGAAATCAATATGATCGTTTCTGa                                                                                                                                                                                                                                                                                                     >  1:111424‑M2/232‑301 (MQ=255)`  
           `atctgatactattattgcccttgagcgtaatcagcaaggcgatatgcctaaccttgtcctcgttcgtattctcaagtgccgctttactggtgatactggtatcgctggctacatggaatacaacaaggaaaccggatggcttgaaccatcaagttactcaggggaagaagagtcacactcagagtcaactgactggtccaacgacactgacttctgacaggattcttgaCAGTTGTTTCATATGAAGAGATTGTTAAGTCACGATAATC‑AATAGGAGAAATCAATATGATCGTTTCTGACa                                                                                                                                                                                                                                                                                                   >  1:152617‑M2/230‑301 (MQ=255)`  
           `atctgatactattattgcccttgagcgtaatcagcaaggcgatatgcctaaccttgtcctcgttcgtattctcaagtgccgctttactggtgatactggtatcgctggctacatggaatacaacaaggaaaccggatggcttgaaccatcaagttactcaggggaagaagagtcacactcagagtcaacagactggtccaacgacactgacttctgacaggattcttgaCAGTTGTTTCATATGAAGAGATTGTTAAGTCACGATAATC‑AATAGGAGAAATCAATATTATCGTTTCTGACa                                                                                                                                                                                                                                                                                                   >  1:209901‑M2/230‑301 (MQ=255)`  
           `atctgatactattattgcccttgagcgtaatcagcaaggcgatatgcctaaccttgtcctcgttcgtattctcaagtgccgctttactggtgatactggtatcgctggctacatggaatacaacaaggaaaccggatggcttgaaccatcaagttactcaggggaagaagagtcacactcagagtcaacagactggtccaacgacactgacttctgacaggattcttgaCAGTTGTTTCATATGAAGAGATTGTTAAGTCACGATAATC‑AATAGGAGAAATCAATATGATCGTTTCTGACa                                                                                                                                                                                                                                                                                                   >  1:156910‑M2/230‑301 (MQ=255)`  
                     `attattgcccttgagcgtaatcagcaaggcgatatgcctaaccttgtcctcgttcgtattctcaagtgccgctttactggtgatactggtatcgctggctacatggaatacaacaaggaaaccggatggcttgaaccatcaagttactcaggggaagaagagtcacactcagagtcaaccgactggtccaactacactgacttctgacaggattcttgaCAGTTGTTTCATATGAAGAGATTGTTAAGTCACGATAATC‑AATAGTAGAAATCAATATGATCGTTTCTGCCATCGAAGCTaa                                                                                                                                                                                                                                                                                         >  1:3298‑M2/220‑301 (MQ=255)`  
                     `attattgcccttgagcgtaatcagcaaggcgatatgcctaaccttgtcctcgttcgtattctcaagtgccgctttactggtgatactggtatcgctggctacatggaatacaacaaggaaaccggatggcttgaaccatcaagttactcaggggaagaagagtcacactcagagtcaacagactggtccaacgacactgacttctgacaggattcttgaCAGTTGTTTCATATGAAGAGATTGTTAAGTCACGATAATC‑AATAGGAGAAATCAATATGATCGTTTCTGACATCGAAgctca                                                                                                                                                                                                                                                                                         >  1:191051‑M2/220‑299 (MQ=255)`  
                                 `gagcgtaatcagcaaggcgatatgcctaaccttgtcctcgttcgtattctcaagtgccgctttactggtgatactggtatcgctggctacatggaatacaacaaggaaaccggatggcttgatccatcaagttactcaggggaagaagagtcacactcagagtcaacagactggtccaacgacactgacttctgacaggattcttgaCAGTTGTTTCATATGAAGAGATTGTTAAGTCACGATAATC‑AATAAGAGAAATCAATATGATCGTTTCTGACATCGAAGCTAACGCCCTCTTaga                                                                                                                                                                                                                                                                             >  1:100119‑M2/208‑301 (MQ=255)`  
                                  `gggagatgtgtataagagacaggcctaaccttgtcctcgttcgtattctcaagtgccgctttactggtgatactggtatcgctggctacatggaatacaacaaggaaaccggatggcttgaaccatcaagttactcaggggaagaagagtcacactcagagtcaacagactggtccaacgacactgacttctgacaggattcttgaCAGTTGTTTCATATGAATAGATTGTTAAGTCACGATAATC‑AATAGGAGAAATCAATATGATCGTTTCTGACATCGAAGCTAACGCCCTCTTagag                                                                                                                                                                                                                                                                            <  1:182860‑M2/95‑1 (MQ=255)`  
                                  `ggcgtaatcagaaatgcgatatggctaaccgtgtcctcgttcgtattctcaagtgccgctttactggtgatactggtatcgctgtctacatggaatacaacaaggaaaccggatggcttgaacaatcaagttactcaggggaagaagagtcacactcagagtcaacagactggtccaacgacactgacttctgacaggattcttgaCAGTTGTTTCATATGAAGAGATTGTTAAGTCACGATAATC‑AATAGGAGAAAGCAATATGATCGTTTCTGACATCGAAGCTAACGCCCTCTTagag                                                                                                                                                                                                                                                                            <  1:160774‑M2/95‑1 (MQ=255)`  
                                  `cgcttcatctgcaaggctatatgcctaaccttgtcctctttcttattctcaagtgccgctttactggtgatactgttatcgctggctacatggaatacaacaaggaaacaggatggcttgaaccatcaagttactcaggggaagaacagtcacactcagagtcaacatccttgtccaacgacactgacttctgacaggattcttgaCAGTTGTTTCATATGAAGAGATTGTTAAGTCACGATAATC‑AATAGGAGAAATCAATATGATCGTTTCTGACATCGAAGCTAACGCCCTCTTagag                                                                                                                                                                                                                                                                            <  1:48709‑M2/95‑1 (MQ=255)`  
                                  `agcgtaatcagccaggagatatgcctaaccttgtcctcgttcgtgttctcacgggccgctttactggtgatactggtaacgctggctacatggaatacaacaaggaaaccggatggcttgaaccatcaagttactcaggggaagaagagtcacactcagagtcaacagactggtccaacgacactgacttctgacaggattcttgaCAGTTGTTTCATATGAAGAGATTGTTAAGTCACGATAATC‑AATAGGAGAAATCAATATGATCGTTTCCGACATCGAAGCTAACGCCCTCTTagag                                                                                                                                                                                                                                                                            <  1:281448‑M2/95‑1 (MQ=255)`  
                                  `agcgtaatcagcaaggcgatatgcctaaccttgtcctagttcgtattctcaagtgccgctttactggtgatactggtatcgctggctacatggaatacaacaaggaaaccggatggcttgaaccatcaagttactcaggggaagaagagtcacactcagagtcaacagactggtccaacgacactgacttctgacaggattcttgaCAGTTGTTTCATATGAAGAGATTGTTAAGTCACGATAATC‑AATAGGAGAAATCAATATGATCGTTTCTGACATCGAAGCTAACACCCTCTTagag                                                                                                                                                                                                                                                                            <  1:106603‑M2/95‑1 (MQ=255)`  
                                    `cgtaatcagcaaggcgatatgcctaaccttgtcctcgttcgtattctcaagtgccgctttacttgtgatactggtatcgctggctacatggaatacaacaaggaaaccggatggcttgaaccatcaagttactcaggggaagaagagtcacactcagagtcaacagactggtccaacgacactgacttctgacagtattcttgaCAGTTGTTTCATATGAAGAGATTGTTAAGTCACGATAATC‑AATAGGAGAAATCAATATGATCGTTTCTGACATCGAAGCTAACGCCCTCTTagagag                                                                                                                                                                                                                                                                          <  1:163506‑M2/97‑1 (MQ=255)`  
                                    `cgtaatcagcaaggcgatatgcctaaccttgtcctcgttcgtattctcaagtgccgctttactggtgatactggtatcgctgactacatggaatacaacaaggaaaccggatggcttgaaccatcaagttactcaggggaagaagagtcacactcagagtcaacagactggtccaacgacactgacttctgacaggattcttgaCAGTTGTTTCATATGAAGAGATTGTTAAGTCACGATAATC‑AATAGGAGAAATCAATATGATCGTTTCTGACATCGAAGCTAACGCCCTCTTagagag                                                                                                                                                                                                                                                                          <  1:286454‑M2/97‑1 (MQ=255)`  
                                                      `atgcctaaccttgtcctcgttcgtattctcaagtgccgctttactggtgatactggtatcgctggctacatggaatacaacaaggaaaccggatggcttgaaccatcaagttactcaggggaagaagagtcacactcagagtcaacagactggtccaacgacactgacttctgacaggattcttgaCAGTTGTTTCATATGAAGAGATTGTTAAGTCACGATAATC‑AATAGGAGACATCAATATGATCGTTTCTGACATCGAAGCTAACGCCCTCTTAGAGAGCGTCACTAAGTTCCAC‑Tg                                                                                                                                                                                                                                                       >  1:147831‑M2/187‑301 (MQ=255)`  
                                                       `tgcctaaccttgtccgcgttagtattctcaagtgccgctttactggtgatactggtatcgctggctacatggaataaaacaaggaaaccggatggcttgaaccatcaagttactcaggggaagaagagtcacactcagagtcaacagactggtccaacgacactgacttctgacaggattcttgaCAGTTGTTTCATATGAAGAGATTGTTAAGTCACGATAATC‑AATAGGAGAAATCAATATGATCGTTTCTGACATCGAAGCTAACGCCCTCTTAGAGAGCGTCACTAAGTTCCAC‑TGc                                                                                                                                                                                                                                                      <  1:217969‑M2/116‑1 (MQ=255)`  
                                                          `ctaaccttgtcctcgttcgtattctcaagtgccgctttactggtgatactggtatcgctggctacatggaatacaacaaggaaaccggatggcttgaaccatcaagttactcaggggaagaagagtcacactcagagtcaacagactggtccaacgacactgacttctgacaggattcttgaCAGTTGTTTCATATGAAGAGATTGTTAAGTCACGATAATC‑AATAGGAGAAATCAATATGATCGTTTCTGACATCGAAGCTAACGCCCTCTTAGAGAGCGTCACTAAGTTCCAC‑TGCggg                                                                                                                                                                                                                                                   <  1:75141‑M2/119‑1 (MQ=255)`  
                                                             `accttgtcgtcgttcgtattctcaagtgccgctttactggtgatactggtatcgctggctacatggaatacaacacggaaaccggatggcttgaaccatcaagttactcaggggaagaagagtcacactcacagtcaacagactggttcaccgacactgccttctgacaggattcttgaCAGTTGTCTCATATGATGAGATTGTTAAATCCAGATAATC‑AATATGCGAATTCAATATGATCGGTTTTGATATCTAAGCCAACGCCCTATTAGCAAACTTCCCTAAGTTCCACTTgcgggtt                                                                                                                                                                                                                                                 >  1:322641‑M2/180‑299 (MQ=255)`  
                                                              `ccttgtcctcgttcgtattctcaagtgccgctttactggtgatactggtatcgctggctacatggaatacaacaaggaaaccggatggcttgaaccatcaagttactcaggggaagaagagtcacactcagagtcaacagactggtccaacgacactgacttctgacaggtttcttgaCAGTTGTTTCATATGAAGAGATTGTTAAGTCACGATAATC‑AATAGGAGAAATCAATATGATCGTTTCTGACATCGAAGCTAACGCCCTCTTAGAGAGCGTCACTAAGTTCCAC‑TGCGGGGTTa                                                                                                                                                                                                                                               >  1:206892‑M2/179‑301 (MQ=255)`  
                                                              `cctggtcctcgttagtattctcaagtgccgctttactggtgatactggtatcgctggctacatggaatacaacaaggaaaccggatggcttgaaccatcaagttactcaggggaagaagagtcacactcagagtcaacagactggtccaacgacactgacttctgacaggattcttgaCAGTTGTTTCATATGAAGAGATTGTTAAGTCACGATAATC‑AATAGGAGAAATCAATATGATCGTTTCTGACATCGAAGCTAACGCCCTCTTAGAGAGCGTCACTAAGTTCCAC‑TGCGGGGTTa                                                                                                                                                                                                                                               <  1:26210‑M2/123‑1 (MQ=255)`  
                                                                 `tgtcctcgttcgtattctcaagtgccgctttactggtgatactggtatcgctggctacatggaatacaacaaggaaaccggatggcttgaaccatcaagttactcaggggaagaagagtcacactcagagtcaacagactggtccaacgacactgacttctgacaggattcttgaCAGTTGTTTCATATGAAGAGATTGTTAAGTCACGATAATC‑AATAGGAGAAATCAATATGATCGTTTCTGACATCGAAGCTAACGCCCTCTTAGAGAGCGTCACTAAGTTCCAC‑TGCGGGGTTATCt                                                                                                                                                                                                                                            <  1:38488‑M2/126‑1 (MQ=255)`  
                                                                  `gtcctcgttcgtattctcaagtgccgctttactggtgatactggtatcgctggctacatggaatacaacaaggaaaccggatggcttgaaccatcaagttactcaggggaagaagagtcaccctcagagtcaacagactggtacaacgacactgacttctgacaggattcttgaCAGTTGTTTCATATGAAGAGATTGTTAAGTCACGATAATC‑AATTGGAGAAATCAATATGATCGTTTCTGACATCGAAGCTAACGCCCTCTTAGAGAGCGTCACTCAGTTCCAC‑TGCGGGGTTATctt                                                                                                                                                                                                                                           >  1:279652‑M2/175‑300 (MQ=255)`  
                                                                  `gtcctcgttcgtattctcaagtgccgctttactggtgatactggcatcgctggctacatggaatacaacaaggaaaccggatggcttgaaccatcaagttactcaggggaagaagagtcacactcagagtcaacagactggtccaacgacactgacttctgacaggattcttgaCAGTTGTTTCATATGAAGAGATTGTTAAGTCACGATAATC‑AATAGGAGAAATCAATATGATCGTTTCTGACATCGAAGCTAACGCCCTCTTAGAGAGCGTCACTAAGTTCCAC‑TGCGGGGTTATCTa                                                                                                                                                                                                                                           >  1:13658‑M2/175‑301 (MQ=255)`  
                                                                    `gcctaaccttgtcctcgttcgtattctcaagtgccgctttactggtgatactggtatcgctggctacatggaatacaacaaggaaaccggatggcttgaaccatcaagttactcaggggaagaagagtcacactcagagtcaacagactggtccaactaaactgacttttgaCAGTTGTTTCATATGAAGAGATTGTTAAGTCACGATAATC‑AATAGGAGAAATCAATATGATCGTTTCTAACATCGAAGCTAACGCACTCTTAGAGAGCGTCACTAAGTTCCAC‑TGCGGGGTTATCTACg                                                                                                                                                                                                                                         >  1:337032‑M2/173‑301 (MQ=255)`  
                                                                      `tcgttggtatgctcaagggcagcttgacgagtgatactggtatcggtaactacatggaagacaacaagaaaactagatagcttgaaccatcaagttactcaagggaagaacggtcacactcagagtcaacaagctggtccaacgacactagctactgacaggattctggaCAATTATTTCATATGAAGAGATTGTTGAGTCACGATGATC‑AATAGGAGAAATCAATATGATCGTTTCTGACATCGAAGCTAACGCCCTCTTAGAGAGCGTCACTAAGTTCCAC‑TGCAGGGTTATCTACGac                                                                                                                                                                                                                                       <  1:340078‑M2/131‑1 (MQ=255)`  
                                                                      `tcgttcgtattctcaagtgccgctttactggtgatactggtatcgctggctacatggaatacaacaaggaaaccggatggcttgaaccatcaagttactcaggggaagaagagtcacgctcagagttaacagactggtccaacgacactgacttctgacaggattcttgaCAGTTGTTTCATATGAAGAGATTGTTAAGTCACGATAATC‑AATAGGAGAAATCAATATGATCGTTTCTGACATCGAAGCTAACGCCCTCTTAGAGAGCGTCACTAAGTTCAAC‑TGCGGGGTTATCTACGac                                                                                                                                                                                                                                       <  1:145745‑M2/131‑1 (MQ=255)`  
                                                                       `cgttcgtattctcaagtgccgctttactggtgatactggtatcgctggctacatggaatacaacaaggaaaccggatggcttgaaccatcaagttactcaggggaagaagagtcacactcagagtcaacagactggtccaacgacactgacttctgacaggattcttgaCAGTTGTTTCATATGAAGAGATTGTTAAGTCACGATAATCAAACAGGAGAAATCAATATGATCGTTTCTGACATCGAAGCTAACGCCCTCTTAGAGAGCGTCACTAAGTTCCAC‑TGCGGGGTTATCTACGac                                                                                                                                                                                                                                       <  1:203973‑M2/132‑1 (MQ=255)`  
                                                                            `ttattctcaagtgccgctttactggtgatactggtatcgctggctacatggaatacaacaaggaaaccggatggcttgaaccatcaagttactcaggggaagaagagtcacactcagagtcaacagactggtccaacgacactgacttctgacaggattcttgaCAGTTGTTTCATATGAAGAGATTGTTAAGTCACGATAATC‑AATAGGAGAAATCAATATGATCGTTTCTGACATCGAAGCTAACGCCCTCTTAGAGAGCGTCACTAAGTTCCAC‑TGCGGGGTTATCTACGACTACTcc                                                                                                                                                                                                                                 <  1:187306‑M2/137‑1 (MQ=255)`  
                                                                              `attctcaagtgccgctttactagtgatacttggaacgctggctacatggaatacaacaaggaaaccggatggcttgaaccatcaagttactcaggggaagaagagtcacactcagagtcaacagactggtccaacgacactgacttctgacaggtttcttgaCAGTTGTTTCATATGAAGATATTGTTAAGTCACGATAATC‑AATAGGAGAAATCAATATGATCGTTTCTGACATCGAAGCTAACGCCCTCTTAGAGAGCGTCACTAAGTTCCAC‑TGCGGGGTTATCAACGACTACTCCAc                                                                                                                                                                                                                               <  1:313296‑M2/139‑1 (MQ=255)`  
                                                                                 `gatgtgtataagagacagggtgatactggtatcgctggctacatggaatacaacaaggaaaccggatggcttgaaccatcaagttactcaggggaagaagagtcacactcagagtcaacagactggtccaaagacactgacttctgacaggattcttgaCAGTTGTTTCATATGAAGAGATTGTTAAGTCACGATAATC‑AATAGGAGAAATCAATATGATCGTTTCTGACATCGAAGCTAACGCCCTCTTAGAGAGCGTCACTAAGTTCCAC‑TGCGGGGTTATCTACGACTACTACACCGc                                                                                                                                                                                                                            <  1:272760‑M2/142‑1 (MQ=255)`  
                                                                                 `ctcaagtgccgctttactggtgatactggtatcgctggctacatggaatacaacaaggaaaccggatggcttgaaccatcacgttactcaggggaagaagagtcacactcagagtcaacagactggtccaacgacactgacttctgacaggattcttgaCAGTTGTTTCATATGAAGAGATTGTTAAGTCACGATAATC‑AATAGGAGAAATCAATATGATCGTTTCTGACATCGAAGCTAACGCCCTCTTAGAGAGCGTCACTACGTTCCAC‑TGCGGGGTtatctacgactacctgtctct                                                                                                                                                                                                                            >  1:68534‑M2/160‑293 (MQ=255)`  
                                                                                   `gagatgggtataagagacagatactggtatagctggctacatggaatacaacaaggaaaacggatggcttgaaccatcaagttactcaggggaagaagagtcacactcagagtcaacagactggtccaacgacactgacttctgacaggattcttgaCAGTTGTTTCATATGAAGAGATTGTTAAGTCACGATAATA‑AATAGGAGAAATCAATATGATCGTTTCTGACATCGAAGCTAACGCCCTCTTAGAGAGCGTCACTAAGTTCCAC‑TGCGGGGTTATCTACGACTACTCCACAGCTg                                                                                                                                                                                                                          <  1:81341‑M2/144‑1 (MQ=255)`  
                                                                                           `gctttactggtgatactggtatcgctggctacatggaatacaacaaggaaaccggatggcttgaaccatcaagttactcaggggaagaagagtcacactcagagtcaacagactggtccaacgacactgacttctgacaggattcttgaCAGTTGTTTCATATGAAGAGATTGTTAAGTCACGATAATC‑AATAGGAGAAATCAATATGATCGTTTCTGACATCGAAGCTAACGCCCTCTTAGAGAGCGTCACTAAGTTCCAC‑TGCGGGGTTATCTACGACTACTCCACCGCTGAGTACGTa                                                                                                                                                                                                                  <  1:130915‑M2/152‑1 (MQ=255)`  
                                                                                             `tttactggtgatactggtatcgctggctacatggaatacaacaaggaaaccggatggcttgaaccatcaagttactcaggggaagaagagtcacactcagagtcaacagactggtccaacgacactgacttctgacaggattcttgaCAGTTGTTTCATATGAAGAGATTGTTAAGTCACGATAATC‑AATAGGAGAAATCAATATGATCGTTTCTGACATCGAAGCTAACGCCCTCTTAGAGAGCGTCACTAAGTTCCAC‑TGCGGGGTTATCTACGACTACTCCACCGCTGAGTACGTAAg                                                                                                                                                                                                                <  1:219910‑M2/154‑1 (MQ=255)`  
                                                                                                `actggtgatactggtatcgctggctacatggaatacaacaaggaaaccggatggcttgaaccatcaagttactcaggggaagaagagtcacactcagagtcaacagactggtccaacgacactgacttctgacaggattcttgaCAGTTGTTTCATATGAAGAGATTGTTAAGTCACGATAATC‑AATAGGAGAAATCAATATGATCGTTTCTGACATCGAAGCTAACGCCCTCTTAGAGAGCGTCACTAAGTTCCAC‑TGCGGGGTTATCTACGACTACTCCACCGCTGAGTCCGTAAGCTa                                                                                                                                                                                                             >  1:201425‑M2/145‑301 (MQ=255)`  
                                                                                                   `ggtgatactggtatcgctggctacatggaatacaacaaggaaaccggatggcttgaactatcaagttactccggggaagaagagtcacactccgagtcaacagactggtccaacgacattgacttctgaccggtttcttgaCAGTTGTTTCATATGAAGAGATTGTTAAGTCACGATAATC‑AATAGGAGAAATCACTATGATCGTTTCTGACATCGAAGCTAACGCCCTCTTAGAGAGCGTCACTAAGTTCCAC‑TGCGGGGGTATCTACGACTACTCCACCGCTGAGTAAGTaagctgtct                                                                                                                                                                                                          >  1:197642‑M2/142‑297 (MQ=255)`  
                                                                                                   `ggtgatactggtatcgctggctacatggaatacaacaaggaaaccggatggcttgaaccatcaagttactcaggggaagaagagtcatactcagagtcaacagactggtccaacgacactgacttctgacagtattcttgaCAGTTGTTTCATATGAAGAGATTGTTAAGTCACGATAATC‑AATAGGAGAAATCAATATGATCGTTTCTGACATCGAAGCTAACGCCCTCTTAGAGAGCGTCACTAAGTTCCAC‑TGCGGGGTTATCTACGACTACTCCACCGCTGAGTACGTAAGCTACCg                                                                                                                                                                                                          <  1:166695‑M2/160‑1 (MQ=255)`  
                                                                                                       `atactggtatcgctggctacatggaatacaacaaggaaaccggatggcttgaaccaccaagctactcaggggaagaagagtcacactcagagtcaacagactggtccaacgacaacgacttctgacaggattcttgaCAGTTGTTTCATATGAAGAGATTGTTAAGTCACGATAACC‑AACAGGAGAAATCAATATGATCGTTTCTGACATCGAAGCTAACGCCCTCTTAGAGAGCGTCACTAAGTTCCAC‑TGCGGGGTTATCTACGACTACTCCACCGCTGAGTACGTAAGCCACCGTACg                                                                                                                                                                                                      >  1:172320‑M2/138‑301 (MQ=255)`  
                                                                                                             `gctcggagatgtgtataagagacagcaaggaaaccggatggcttgaaccatcaagttactcatgggaagaagagtcacactcagagtcaacagactggtccaacgacactgacttctgacaggattcttgaCAGTTGTTTCATATGAAGAGATTGTTAAGTCACGATAATC‑AATAGGAGAAATCAATATGATCGTTTCTGACATCGAAGCTAACGCCCTCTTAGAGAGCGTCACTAAGTTCCAC‑TGCGGGGTTATCTACGACTACTCCACCGCTGAGTACGTAAGCTACCGTCCGAGTGAc                                                                                                                                                                                                <  1:229440‑M2/170‑1 (MQ=255)`  
                                                                                                                         `acatggaatacaacaaggaaaccggatggcttgaaccatcaagttactcaggggaagaagagtcacactcagagtcaacagactggtccaacgacactgacttctgacaggattcttgaCAGTTGTTTCATATGAAGAGATTGTTAAGTCACGATAATC‑AATAGGAGAAATCAATATGATCGTTTCTGACATCGAAGCTAACGCCCTCTTAGAGAGCGTCACTAAGTTCCAC‑TGCGGGGTTATCTACGACTACTCCACCGCtgagtacgtaagctaccgtcctgtctcttatacacatctc                                                                                                                                                                                    >  1:169689‑M2/120‑282 (MQ=255)`  
                                                                                                                              `gaatacaacaaggaaaccggatggcttgaaccatcaagttactcaggggaataagagtcacactcagagtcaacagactggtccaacgacactgacttctgacaggattcttgaCAGTTGTTTCATATGAAGAGATTGTTAAGTCACGATAATC‑AATAGGAGAAATCAATATGATCGTTTCTGACATCGAAGCTAACGCCCTCTTAGAGAGCGTCACTAAGTTCCAC‑TGCGGTGTTATCTACGACTACTCCACCGCTGAGTACGTAAGCTACCGTCCGAGTGACTTCGGTGCGTATCTGGa                                                                                                                                                                               >  1:238182‑M2/115‑301 (MQ=255)`  
                                                                                                                                  `acaacaaggaaaccggatggcttgaaccatcaagttactcaggggaagaagagtcacactcagagtcaacagactggtccaacgacactgacttctgacaggattcttgaCAGTTGTTTCATATGAAGAGATTGTTAAGTCACGATAATC‑AATAGGAGAAATCAATATGATCGTTTCTGACATCGAAGCTAACGCCCTCTTAGAGAGCGTCACTAAGTTCCAC‑TGCGGGGTTATCTACGACTACTCCACCGCTGAGTACGTAAGCTACCGTCCGAGTGACCTCGGAGCTTTTCTggatctg                                                                                                                                                                           >  1:310146‑M2/111‑298 (MQ=255)`  
                                                                                                                                                    `ggcttgaaccatcaagttactcatgggaagaagagacacactcagagtcaacagactggtccaacgacactgacttcagacaggaatcttgaCAGTTGTTTCATATGAAGAGATTGTTAAGTCACGATAATC‑AATAGGAGAAAACAATATGATCGTTCCTGACATCGAAGCTACCGCCCTCTTAGAGAGCGTCACTAAGTTCCAC‑TGCTGGGTTAACTACGACTACTACACCGCTGAGTACGTAAGCTACCGTCCGAGTGACTTCGGTGCGTAACTGGATGCGCTGGAAGACGAGGctgtg                                                                                                                                                         >  1:155015‑M2/93‑299 (MQ=255)`  
                                                                                                                                                     `gcttgaaccatcaagttactcaggggaagaagagtcacactcagagtcaacagactggtcccacgacactgacttctgacaggattcttgaCAGCTGTTTCATATGAAGAGATTGTTAAGTCACGTTAATC‑TATAGGAGAAATCAATATGATCGTTTCTGACATCGAAGCTAACGCCCTCTTAGAGAGCGTTACTAAGTTCCAC‑TGCGGGGGTATCTACGACTACTCCAACGCTGAGTACGTAAGCTACAGTCCGAGTGACTTCGGTGCGTATCTGGATGTGATGGAAGACGCGGTTGAAc                                                                                                                                                        >  1:72212‑M2/92‑301 (MQ=255)`  
                                                                                                                                                                `ggcttgaaccatcaagttactcaggggaagaagagtcacactcagagtcaacagactggtccaacgacactgacttttgaCAGTTGTTTCATATGAAGAGATTGTTAAGTCACGATAATC‑AATAGTAGAAATCAATATGATCGTTTCTGACATCGAAGCTAACGCCCTCTTAGAGAGCGTCACTAAGTTCCAC‑TGCGGGGTTATCTACGACTACTCCACCGCTGAGTACGTAAGCTACCGTCCGAGTGACTTCGGTGCGTATCTGGATTCGCTGGAAGCCGAGGTTGCACGAGGAGGTCtt                                                                                                                                             >  1:51748‑M2/81‑301 (MQ=255)`  
                                                                                                                                                                 `aagttactcaggggaagaagagtcacactcagagtcaacatactggtccaacgacactgacttctgacaggattcttgaCAGTTGTTTCATATGAAGAGATTGTTAAGTCACGATAATC‑AATAGGAGAAATCAATATGATCGTTTCTGACATCGAAGCTAACGCCCTCTTAGAGAGCGTCACTAAGTTCCAC‑TGCGGGGTTATCTACGACTACTCCACCGCTGAGTACGTAAGCTAACGTCCGAGTGACTTCGGTGCGTATCTGGATGCGCTGGAAGCCGAGGTTGCACGAGGTGGCcttt                                                                                                                                            >  1:250588‑M2/80‑300 (MQ=255)`  
                                                                                                                                                                    `ttactcatgggaagaagagtcacactcagagtcaacagactggtccaacgacactgacttctgacaggattcttgaCAGTTGTTTCATATGAAGAGATTGTTAAGTCACGATAATC‑AATAGGAGAAATCAATATGATCGTTTCTGACATCGAAGCTAACGCCCTCTTAGAGAGCGTCACTAAGTTCCAC‑TGCGGGGTTATCTACGACTACTCCACCGCTGAGTACGTAAGCTACCGTCCGAGTGACTTCGGTGCGTATCTGGATGCGCTGGAAGCCGAGGTTGCACGAGGCGGTCTTATtg                                                                                                                                         <  1:40790‑M2/225‑1 (MQ=255)`  
                                                                                                                                                                    `tgggctaggacatgtgtaaaagaaacagtggtcaactgactggtccaacgacactgacttctgacaggatttttgaCAGTTGTTTCATATGAAGAGATTGTTAAGTCACGATAATG‑AATAGGAGAAATCAAGATGATCGGTTCTGAAAGCGAAGCTAACGCACTCTTAGAGAGCGTCACTAAGTTCCAC‑TGCGGGGTTATCTACGACTACTCCACCGATGAGTACGTAAGCTACCGTCGGAGTGACTTCGGTGCGTATCTGGATGCGCTGGAAGCCGAGGTTGCACGAGGCGGTCTTATtg                                                                                                                                         <  1:55677‑M2/225‑1 (MQ=255)`  
                                                                                                                                                                    `cggagatgttgataagaaacagcactcagagtcaacagactggtccaacgacactgacttcttacaggattcttgaCAGTTGTTTCATATGAAGAGATTGTTAAGTCACGATAATC‑AATAGTAGAAATCAATATGATCGTTTCTGACATCGAAGCTAACGCCCTCTTAGAGAGCGTCACTAAGTTCCAC‑TGCGGGGTGATCTACGACTACTCCACCGCTGAGTACGTAAGCTACCGTCCGAGTGACTTCGGTGCGTATCTGGATGCGCTGGAAGCCGAGGTTGCACGAGGCGGTCTTATtg                                                                                                                                         <  1:254592‑M2/225‑1 (MQ=255)`  
                                                                                                                                                                         `caggggaagaagagtcacactcagagtcaaaagactggtccaacgacactgacttctgacaggtttcttgaCAGTTGTTTCATATGAAGAGATTGTTAAGTCACGATATTC‑AATAGGAGAAATCAATATGATCGTTTCTGACATCGAAGCTAACGCCCTCTTAGAGAGCGTCACTAAGTTCCAC‑TGCGGGGTTATCTACGACTACTCCACCGCTGAGTACGTAAGCTACCGTCCGAGTGACTTCGGTGCGTATCTGGATGCGCTGGAAGCCGAGGTTGCACGAGGCGGTCTTATTGTGTTc                                                                                                                                    <  1:270918‑M2/230‑1 (MQ=255)`  
                                                                                                                                                                           `ggggaagaagagtcacactcagagtcaacagactggtccaacgacactgacttctgacaggattcttgaCAGTTGTTTCATATGAAGAGATTGTTAAGTCACGATAATC‑AATAGGAGAAATCAATATGATCGTTTCTGACATCGAAGCTAACGCCCTCTTAGAGAGCGTCACTAAGTTCCAC‑TGCGGGGTTATCTACGACTACTCCACCGCTGAGTACGTAAGCTACCGTCCGAGTGACTTCGGTGCGTATCTGGATGCGCTGGAAGCCGAGGTTGCACGAGGCGGTCTTATTGTGTTCca                                                                                                                                  >  1:258542‑M2/70‑301 (MQ=255)`  
                                                                                                                                                                           `ggggaagaagagtcacactcagagtcaacagactggtccaacgacactgacttctgacaggattcttgaCAGTTGTTTCATATGAAGAGATTGTTAAGTCACGATAATC‑AATAGGAGAAATCAATATGATCGTTTCTGACATCGAAGCTAACGCCCTCTTAGAGAGCGTCACTAAGTTCCAC‑TGCGGGGTTATCTACGACTACTCCACCGCTGAGTACGTAAGCTACCGTCCGAGTGACTTCAGTGCGTATCTGGATGCGCTGGAAGCCGAGGTTGCACGAGGCGgtcttattgctgtctt                                                                                                                                  >  1:90277‑M2/70‑294 (MQ=255)`  
                                                                                                                                                                             `aagttactcaggggaagaagagtcacactcagagtcaacagactggtccaacgacactgacttttgaCAGTTGTTTCATATGAAGAGATTGTTAAGTCACGATAATC‑AATAGGAGAAATCAATATGATCGTTTCTGACATCGAAGCTAACGCCCTCTTAGAGAGCGTCACTAAGTTCCAC‑TGCGGGGTTATCTACGACTACTCCACCGCTGAGTACGTAAGCTACCGTCCGAGTGACTTCGGTGCGTATCTGGATGCGCTGGAAGCCGAGGTTGCACGAGGCGgtattattgtgctgtctc                                                                                                                                >  1:276197‑M2/68‑294 (MQ=255)`  
                                                                                                                                                                              `gaagaagagtcacactcagagtcaacagacttgtccaacgacactgacttctgacaggattcttgaCAGTTGTTTCATATGAAGAGATTGTTAAGTCATGATAATC‑AATAGGAGAAATCAATATGATCGTTTCTGACATCGAAGCTAACGCCCTCTTAGAGAGCGTCACTAAGTTCCAC‑TGCGGTGTTATCTACGACTACTCCACCGCTGAGTACGTAAGCTACCGTCCGAGTGACTTCGGTGCGTATCTGGATGCGCTGGAAGCCGAGGTTGCACGAGGCGGTCTTATTGTGTTCCACaa                                                                                                                               >  1:85774‑M2/67‑301 (MQ=255)`  
                                                                                                                                                                              `gaagaagagtcacactcagagtcaacagactggtccaacgacactgacttctgacaggattcttgaCAGTTGTTTCATATGAAGAGATTGTTAAGTCACGATAATC‑AATAGGAGAAATCAATATGATCGTTTCTGACATCGAAGCTAACGCCCTCTTAGAGAGCGTCACTAAGTTCCAC‑TGCGGGGTTATCTACGACTACTCCACCGCTGAGTACGTAAGCTACCGTCCGAGTGACTTCGGTGCGTATCTGGATGCGCTGGAAGCCGAGGTTGCACGAGGCGGTCTTATTGTGTTCCACaa                                                                                                                               <  1:306070‑M2/235‑1 (MQ=255)`  
                                                                                                                                                                              `gaagaagagtcacactcagagtcaacagactggtccaacgacactgacttctgacaggattcttgaCAGTTGTTTCATATGAAGAGATTGTTAAGTCACGATAATC‑AATAGGAGAAATCAATATGATCGTTTCTGACATCGAAGCTAACGCCCTCTTAGAGAGCGTCACTAAGTTCCAC‑TGCGGGGTTATCTACGACTACTCCACCGCTGAGTACGTAAGCTACCATCCGAGTGACTTCGGTGCGTATCTGGATGCGCTGGAAGCCGAGGTTGCACGAGGCGGTCTTATTGTGTTCCACaa                                                                                                                               <  1:193044‑M2/235‑1 (MQ=255)`  
                                                                                                                                                                                `agaagagtcacactcagagtcaacagactggtccaacgacactgacttctgacaggattcttgaCAGTTGTTTCATATGAAGAGATTGTTAAGTCACGATAATC‑AATAGGAGAAATCAATATGCTCGTTTCTGACATCGAAGCTAACGCCCTCTTAGAGAGCGTCACTAAGTTCCAC‑TGCGGGGTTATCTACGACTACTCCACCGCTGAGTACGTAAGCTACCGTCCGAGTGACTTCGGTGCGTATCTGGATGAGCTGGACGCCGAGGTCGCACGAGGCGGCCTTATTGTGTTCCACAACg                                                                                                                             >  1:227432‑M2/65‑301 (MQ=255)`  
                                                                                                                                                                                        `cacactcagagtcaacagactggtccaacgacactgacttctgacaggattcttgaCAGTTGTTTCATATGAAGAGATTGTTAAGTCACGATAATC‑AATAGGAGAAATCAATATGATCGTTTCTGACATCGAAGCTAACGCCCTCTTAGAGAGCGTCACTAAGTTCCAC‑TGCGGGGTTATCTACGACTACTCCACCGCTGAGTACGTAAGCTACCGTCCGAGTGACTTCGGTGCGTATCTGGATGCGCTGGAAGCCGAGGTTGCACTATGCGGTCTTATTGTGTTCCACAACGGTCACAAg                                                                                                                     <  1:133508‑M2/245‑1 (MQ=255)`  
                                                                                                                                                                                              `gctcggagatgtgtataagagacagcactgacttctgacaggattcttgaCAGTTGTTTCATATGAAGAGATTGTTAAGTCACGATAATC‑AATAGGAGAAATCAATATGATCGTTTCTGACATCAAAGCTAACGCCCTCTTAGAGAGCGTCACTAAGTTCCAC‑TGCGGGGTTATCTACGACTACTCCACCGCTGAGTACGTAAGCTACCGTCCGAGTGACTTCGGTGCGTATCTGGATGCGCTGGAAGCCGAGGTTGCACGAGGCGGTCTTATTGTGTTCCACAACGGTCACAAGTATGAc                                                                                                               <  1:174581‑M2/251‑1 (MQ=255)`  
                                                                                                                                                                                                `tgtgtataagagacagccaacgacactgacttctgacaggattcttgaCAGTTGTTTCATATGAAGAGATTGTTAAGTCACGATAAAC‑AATAGGAGAAATCAATATGATCGTTTCTGACATCGAAGCTAACGCCCTCTTAGAGAGCGTCACTAAGTTCCAC‑TGCGGGTTTATCTACGACTACTCCACCGCTGAGTACGTAAGCTACCGTCCGAGTGACTTCGGTGCGTATCTGGATGCGCTGGAAGCCGAGGTTGCACGAGGCGGTCTTATTGTGTTCCACAACGGTCACAAGTATGACGt                                                                                                             <  1:150150‑M2/253‑1 (MQ=255)`  
                                                                                                                                                                                                `gagtcaacagactggtccaacgacactgacttctgacaggattcttgaCAGTTGTTTAATATGAAGAGATTGTTAAGTCACGATAATC‑AATAGGAGAAATCAATATGATCGTTTCTGACATCGAAGCTAACGCCCTCTTAGAGAGCGTCACTAAGTTCCAC‑TGCGGGGTTATCTACGACTACTCCACCGCTGAGTACGTAAGCTACCGTCCGAGTGACTTCGGTGCGTATCTGGATGCGCTGGAAGCCGAGGTTGCACGAGGCGGTCTTATTGTGTTCCACAACGGTCACAAGTATGACGt                                                                                                             <  1:323873‑M2/253‑1 (MQ=255)`  
                                                                                                                                                                                                 `agtcaacagactggtccaacgacactgacttcttacaggattcttgaCAGTTGTTTCATATGAAGAGATTGTTAAGTCACGATAATC‑AATAGGAGAAATCAATATGATCGTTTCTGACATCGAAGCTAACGCCCTCTTAGAGAGCGTCACTAAGTTCCAC‑TGCGGGGTTATCTACGACTACTCCACCGCTGAGTACGTAAGCTACCGTCCGAGTGACTTCGGTGCGTATCTGGATGCGCTGGAAGCCGAGGTTGCACGAGGAGGTCTTATTGTGTTCCACAACGGTcacaagtactgtctc                                                                                                            >  1:288678‑M2/48‑294 (MQ=255)`  
                                                                                                                                                                                                  `gtcaacagactggtccaacgacactgacttctgacaggattcttgaCAGTTGTTTCATATGAAGAGATTGTTAAGTCACGATAATC‑AATAGGAGAAATCAATATGATCGTTTCTGACATCGAAGCTAACGCCCTCTTAGAGAGCGTCACTAAGTTCCAC‑TGCGGGGTTATCTACGACTACTCCACCGCTGAGTACGTAAGCTACCGTCCGAGTGACTTCGGTGCGTATCTGGATGCGCTGGAAGCCGAGGTTGCACGAGGCGGTCTTATTGTGTTCCACAACGGTCACAAGTATGACGTTc                                                                                                           >  1:300845‑M2/47‑301 (MQ=255)`  
                                                                                                                                                                                                        `agactggtccaacgacactgacttctgacaggtttcttgaCAGTTGTTGCATATGAAGAGATTGTTAAGTCACGATAATC‑AATAGGAGAAATCAATATGATCGTTTCTGACATCGAAGCTAACGCCCTCTTAGAGAGCGTCACTAAGTTCCAC‑TGCAGGGTTATCTACGACTACTCCACCGCTGAGTACGTAAGCTACCGTCCGAGTGACTTCGGTGCGTATCTGGATGCGCTGGAAGCCGAGGTTGCACGAGGCGGTCTTATTGTGTTCCACAACGGTCACAAGTATGACGTTCCTGCAt                                                                                                     <  1:212226‑M2/261‑1 (MQ=255)`  
                                                                                                                                                                                                         `gactggtccaacgacactgacttctgacaggattcttgaCAGTTGTTTCATATGAAGAGATTGTTAAGTCACGATAATC‑AATAGGAGAAATCAATATGATCGTTTCTGACATCGAAGCTAACGCCCTCTTAGAGAGCGTCACTAAGTTCCAC‑TGCGGGGTTATCTACGACTACTCCACCGCTGAGTACGTAAGCTACCGTCCGAGTGACTTCGGTGCGTATCTGGATGCGCTGGAAGCCGAGGTGGCACGAGGCGGTCTTATTGTGTTCCACAACGGTCACAAGTATGACGTTCTTGCAtt                                                                                                    >  1:43753‑M2/40‑301 (MQ=255)`  
                                                                                                                                                                                                             `ggtccaacgacactgacttctgacaggattcttgaCAGTTGTTTCATATGAAGAGATTGTTAAGTCACGATAATC‑AATAGGAGAAATCAATATGATCGTTTCTGACATCGAAGCTAACGCCCTCTTAGAGAGCGTCACTAAGTTCCAC‑TGCGGGGTTATCTACGACTACTCCACCGCTGAGTACGTAAGCTACCGTCCGAGTGACTTCGGTGCGTATCTGGATGCGCTGGAAGCCGAGGTTGCACGAGGCGGTCTTATTGTGTTCCACAACGGTCACAAGTATGACGTTCCTGCATTGAcc                                                                                                >  1:78430‑M2/36‑301 (MQ=255)`  
                                                                                                                                                                                                                    `cgacactgacttctgacaggattcttgaCAGTTGTTTCATATGAAGAGATTGTTAAGTCACGATAATC‑AATAGGAGAAATCAATATGATCGTTTCTGACATCGAAGCTAACGCCCTCTTAGAGAGCGTCACTAAGTTCCAC‑TGCGGGGTTATCTACGACTACTCCACCGCTGAGTACGTAAGCTACCGTCAGAGTGACTTCGGTGCGTATCTGGATGCGCTGGAAGCCGAGGTTGCACGAGGCGGTCTTATTGTGTTCCACAACGGTCACAAGTATGACGTTCCTGCATTGACCAAACTgg                                                                                         >  1:158454‑M2/29‑301 (MQ=255)`  
                                                                                                                                                                                                                         `gagatgtgtataagagacagtgaCAGTTGTTTCATATGAAGAGATTGTTAAGTCACGATAATC‑AATATGAGAAATCAATATGATCGTTTCTGACATCGAAGCTAACGCCCTCTTAGAGAGCGTCACTAAGTTCCAC‑TGCGGGGTTATCTACGACTACTCCACCGCTGAGTACGTAAGCTACCGTCCGAGTGACTTCGGTGCGTATCTGGATGCGCTGGAAGCCGAGGTTGCACGAGGCGGTCTTATTGTGTTCCACAACGGTCACAAGTATGACGTTCCTGCATTGACCAAACTGGCAAAg                                                                                    <  1:115329‑M2/278‑1 (MQ=255)`  
                                                                                                                                                                                                                            `taagagacagggattcttgaCATTTGTTTCATATGAAGAGATTGTTAAGTCACAATAATC‑AATAGGAGAAATCAATATGATCGTTTCTGACATCGAAGCTAACGCCCTCTTAGAGAGCGTCACTAAGTTCCAC‑TGCGGGGTTATCTACGACTACTCCACCGCTGAGTACGTAAGCTACCGTCCGAGTGACTTCGGTGCGTATCTGGATGCGCTGGAAGCCGAGGTTGCACGAGGCGGTCTTATTGTGTTACACAACGGTCACAAGTATGACGTTCCTGCATTGACCAAACTGGCAAAGTTg                                                                                 <  1:34147‑M2/281‑1 (MQ=255)`  
                                                                                                                                                                                                                                     `aggattcttgaCAGTTGTTTCATATGAAGAGATTGTTAAGTCACGATAATC‑AAGAGGAGAAATCAATATGATCGTTTCTGACATCGAAGCTAACGCCCTCTTAGAGAGCGTCACTAAGTTCCAC‑TGCGGGGTTATCTACGACTACTCCACCGATGAGTACGTAAGCTACCGTCCGAGTGACTTCGGTGCGTATCTGGATGCGCTGGAAGCCGAGGTTGCACGAGGCGGTCTTATTGTGTTCCACAACGGTCACAAGTATGACGTTCCTGCATTGACCAAACTGGCAAAGTTGCAATTGAAc                                                                        <  1:62582‑M2/290‑1 (MQ=255)`  
                                                                                                                                                                                                                                           `cttgaCAGGTGTTTAAGATGAGGAGATTGTTAAGTCACGAGAATC‑AATAGGAGAAATCAATATGCTCGTTTCTGACATCGAAGGTAACGCCCTCGTAGAGAGCGTCACTAAGTGCCAC‑TGGGGGGTTATCTACGCCGAGTCCACCGCTGAGTACGTAAGCTACTGTCCGAGTGACTTCGGTGCGTATCTGGTTGCGCTGGAAGCCGAGGTTGCACGAGGCGGTCTTATTGTGTTCCACAACGGTCACAAGTATGACGTTCCTGCATTGACCAAACTGGCAAAGTTGCGATTGAACCGagag                                                                  <  1:139178‑M2/296‑1 (MQ=255)`  
                                                                                                                                                                                                                                              `gaacgatgaggctcgtaaagaggccacacaGTCACGATAATC‑AATAGGAGAAATCAATATGATCGTTTCTGACATCGAAGCCAACGCCCTCTTAGAGAGCGTCACTAAGTTCCAC‑TGCGGGGTTATCTACGACTACTCCACCGCTGAGTACGTAAGCTACCGTCCGAGTGACTTCGGTGCGTATCTGGATGCGCTGGAAGCCGAGGTTGCACGAGGCGGTCTTATTGTGTTCCACAACGGTCACAAGTATGACGTTCCTGCATTGACCAAACTGGCAAAGTTGCAATTGAACCGAGAGTTc                                                               >  1:126408/30‑301 (MQ=255)`  
                                                                                                                                                                                                                                                 `aGTTGTTTCATATGAAGAGATTGTTAAGTCACGATAATC‑AATAGGAGAAATCAATATGATCGTTTCTGACATCGAAGCTAACGCCCTCTTAGAGAGCGTCACTAAGTTCCAC‑TGCGGGGTTATCTACGACTACTCCACCGCTGAGTACGTAAGCTACCGTCCGAGTGACTTCGGTGCGTATCTGGATGCGCTGGAAGCCGAGGTTGCACGAGGCGGTCTTATTGTGTTCCACAACGGTCACAAGTATGACGTTCCTGCATTGACCAAACTGGCAAAGTTGCAATTGAACCGAGAGTTCCAc                                                            <  1:58630/301‑1 (MQ=255)`  
                                                                                                                                                                                                                                                    `tgttTCATATGAAGAGATTGTTAAGTCACGATAATC‑AATAGGAGAAATCAATATGATCGTTTCTGACATCGAAGCTAACGCCCTCTTAGAGAGCGTCACTAAGTTCCAC‑TGCGGGGTTATCTACGACTACTCCACCGCTGAGTACGTAAGCTACCGTCCGAGTGACTTCGGTGCGTATCTGGATGCGCTGGAAGCCGAGGTTGCACGAGGCGGTCTTATTGTGTTCCACAACGGTCACAAGTATGACGTTCCTGCATTGACCAAACTGGCAAAGTTGCAATTGAACCGAGAGTTCCACCtt                                                         >  1:215164/1‑301 (MQ=255)`  
                                                                                                                                                                                                                                                         `cATATGAAGAGATTGTTAAGTCACGATAATC‑AATAGGAGAAATCAATATGATCGTTTCTGACATCGAAGCTAACGCCCTCTTAGAGAGCGTCACTAAGTTCCAC‑TGCGGGGTTATCTACGACTACTCCACCGCTGAGTACGTAAGCTACCATCCGAGTGACTTCGGTGCGTATCTGGATGCGCTGGAAGCCGAGGTTGCACGAGGCGGTCTTATTGTGTTCCACAACGGTCACAAGTATGACGTTCCTGCATTGACCAAACTGGCAAAGTTGCAATTGAACCGAGAGTTCAACCTTCCTCg                                                    >  1:222396/1‑301 (MQ=255)`  
                                                                                                                                                                                                                                                                 `tcggagatgggtataagagacagaaaTAGGAGAAATCAATAGGATCGTTTCTGACATCGAAGCTAACGCCCTCTTAGAGAGCGTAACTAAGTTCCAC‑TGCGGGGTTATCTACGACTACTCCACCGCTGAGTACGTAAGCTACAGTCCGGGTGACTTCGGTGCGTATCTGGATGCGCTGGAAGCCGAGGTTGCACGAGGCGGTCTTATTGTGTTCCACAACGGTCACAAGTATGACGTTCCTGCATTGACCAAACTGGCAAAGTTGCAATTGAACCGAGAGTTCAACCTTCCTCGTGAGAAc                                             <  1:26515/277‑1 (MQ=255)`  
                                                                                                                                                                                                                                                                   `gaTTGTTAAGTCACGATAATC‑AATAGGAGAAATCAATATGATCGTTTCTTACATCGAAGCTAACGCCCTCTTAGAGAGCGTCACTAAGTTCCAC‑TGCGGGGTTATCTACGACTACTCCACCGCTGAGTACGTAAGCTACCGTCCGAGTGACTTCGGTGCGTATCTGGATGCGCTGGAAGCCGAGGTTGCACGAGGCGGTCTTATTGTGTTCCACAACGGTCACAAGTATGACGTTCCTGCATTGACCAAACTGGCAAAGTTGCAATTGAACCGAGAGTTCCACCTTCCTCGTGAGAACTGt                                          >  1:246510/1‑301 (MQ=255)`  
                                                                                                                                                                                                                                                                       `gTTAAGTCACGATAATC‑AATAGGAGAAATCAATATGATCGTTTCTGACATCGAAGCTAACGCCCTCTTAGAGAGCGTCACTAAGTTCCAC‑TGCGGGGTTATCTACGACTACTCCACCGCTGAGTACGTAAGCTACCGTCCGAGTGACTTCGGTGCGTATCTGGATGCGCTGGAAGCCGAGGTTGCACGAGGCGGTCTTATTGTGTTCCACAACGGTCACAAGTATGACGTTCCTGCATTGACCAAACTGGCAAAGTTGCAATTGAACCGAGAGTTCCACCTTCCTCGTGAGAACTGTAtct                                      >  1:232624/1‑299 (MQ=255)`  
                                                                                                                                                                                                                                                                         `tAAGTCACGATAATC‑AATAGGAGAAATCAATATGATCGTTTCTGACATCGAAGCTAACGCCCTCTTAGAGAGCGTCACTAAGTTCCAC‑TGCGGGGTTATCTACGACTACTCCACCGCTGAGTACGTAAGCTACCGTCCGAGTGACTTCGGTGCGTATCTGGATGCGCTGGAAGCCGAGGTTGCACGAGGCGGTCTTATTGTGTTCCACAACGGTCACAAGTATGACGTTCCTGCATTGACCAAACTGGCAAAGTTGCAATTGAACCGAGAGTTCCACCTTCCTCGTGAGAACTGTATTGac                                    <  1:102540/301‑1 (MQ=255)`  
                                                                                                                                                                                                                                                                         `tAAGTCACGATAATC‑AATAGGAGAAATCAATATGATCGTTTCTGACATCGAAGCTAACGCCCTCTTAGAGAGCGTCACTAAGTTCCAC‑TGCGGGGTTATCTACGACTACTCCACCGCTGAGTACGTAAGCTACCGTCCGAGTGACTTCGGTGCGTATCTGGATGCGCTGGAAGCCGAGGTTGCACGAGGCGGTCTTATTGTGTTCCACAACGGTCACAAGTATGACGTTCCTGCATTGACCAAACTGGCAAAGTTGCAATTGAACCGAGAGTTCCACCTTCCTCGTGAGAACTGTATTGac                                    <  1:297179/301‑1 (MQ=255)`  
                                                                                                                                                                                                                                                                          `caGTCACGATAATCAAACAGGTGCAATCAATATGATCGTTTCTGACATCGAAGCTAACGCCCTCTTAGAGAGCGTCACTAAGTTCCAC‑TGCGGGGTTATCTACGACTACTCCACCGCTGAGTACGTAAGCTACCGTCCGAGTGACTTCGGTGCGTATCTGGATGCGCTGGAAGCCGAGGTTGCACGAGGCGGTCTTATTGTGTTCCACAACGGTCACAAGTATGACGTTCCTGCATTGACCAAACTGGCAAAGTTGCAATTGAACCGAGAGTTCCACCTTCCTCGTGAGAACTGTATTGac                                    <  1:326650/300‑1 (MQ=255)`  
                                                                                                                                                                                                                                                                          `aaGTCACGATAATC‑AATAGGAGAAATCAATATGATCGTTTCTGACATCGAAGCCAACTCCCTCTTAGAGAGCGTCACTAAGTTCCAC‑TGCGGGGTTATCTACGACTACTCCACCGCTGAGTACGTAAGCTACCGTCCGAGTGACTTCGGTGCGTATCTGGATGCGCTGGAAGCCGAGGTTGCACTAGGCGGTCTTATTGTGTTCCACAACGGTCACAAGTATGACGTTCCTGCATTGACCAAACTGGCAAAGTTGCAATTGAACCGAGAGTTCCACCTTCCTCGTGAGAACTGTATTGaca                                   <  1:94534/301‑1 (MQ=255)`  
                                                                                                                                                                                                                                                                           `aGTCACGATAATC‑AATAGGAGAAATCAATATGATCGTTTCTGACATCGAAGCTAACGCCCTCTTAGAGAGCGTCACTAAGTTCTAC‑TGCGGGGTTATCTACGACTACTCCACCGCAGAGTACGTAAGCTACCGTCCGAGTGACTTCGGTGCGTATCTGGATGCGCTGGAAGCCGAGGTTGCACGAGGCGGTCTTATTGTGTTCCACAACGGTCACAAGTATGACGTTCCTGCATTGACCAAACTGGCAAAGTTGCAATTGAACCGAGAGTTCCACCTTACTCGTGAGAACTGTATTGacac                                  <  1:122286/301‑1 (MQ=255)`  
                                                                                                                                                                                                                                                                            `ataagagacagcactaaaggagacacaccatGATCGTTTCTGACATCGAAGCTAACGCCCTCTTAGAGAGCGTCACTAAGTTCCAC‑TGCGGGGTTATCTACGACTACTCCACCGCTGAGTACGTAAGCTACCGTCCGAGTGACTTCGGTGCGTATCTGGATGCGCTGGAAGCCGAGGTTGCACGAGGCGGTCTTATTGTGTTCCACAACGGTCACAAGTATGACGTTCCTGCATTGACCAAACTGGCAAAGTTGCAATTGAACCGAGAGTTCCACCTTCCTCGTGAGAACTGTATTGacac                                  <  1:97228/272‑1 (MQ=255)`  
                                                                                                                                                                                                                                                                             `ttttatgaatcaaacaagataaaCCATTATGATCGTTTCCGACATCGAACCTCACGCCCTTCTAGAGTGCCTTACTAAGTTCCAC‑TGCGGGGTTATCTACGACTACGCCACCGCTGAGTACGTAAGCTACCGTCCGAGTGACTTCGGTGCGTATCTGGATGCGCTGGAAGCCGAGGTTGCACGAGGCGGTCTTATTGTGTTCCACAACGGTCACAAGTATGACGTTCCTGCATTGACCAAACTGGCAAAGTTGCAATTGAACCGTGAGTTCCACCTTCCTCGTGAGAACTGTATTGACAcc                                 <  1:29474/281‑1 (MQ=255)`  
                                                                                                                                                                                                                                                                              `cACGATAATCAAACAGGAGAAACCATTATGATCGTTTCTGACATCGAAGCCAACGCCCTCTTAGAGAGCGTCACTAAGTTCCAC‑TGCGGGGTTATCTACGACTACTCCACCGCTGAGTACGTAAGCTACCGTCCGAGTGACTTCGGTACGTATCTGGATGCGCTGGAAGCCGAGGTTGCACGAGGCGGTCTTATTGTGTTCCACAACGGTCACAAGTATGACGTTCCTGCATTGACCAAACTGGCAAAGTTGCAATTGAACCGAGAGTTCCACCTTCCTCGTGAGAACTGTAtctgtctct                                >  1:29081/1‑293 (MQ=255)`  
                                                                                                                                                                                                                                                                                `cGATAATC‑AATAGGAGAAATCAATATGATCGTTTCTGACATCGAAGCTAACGCCCTCTTAGAGAGCGTCACTAAGTTCCAC‑TGCGGGGTTATCTACGACTACTCCACCGCTGAGTACGTAAGCTACCGTCCGAGTGACTTCGGTGCGTATCTGGATGCGCTGGAAGCCGAGGTTGCACGAGGCGGTCTTATTGTGTTCCACAACGGTCACAAGTATGACGTTCCTGCATTGACCAAACTGGCAAAGTTGCAATTGAACCGAGAGTTCCACCTTCCTCGTGAGAACTGTATTGACACCCttt                             >  1:141593/1‑300 (MQ=255)`  
                                                                                                                                                                                                                                                                                  `gtaatcaaacaGGAGAAACCATTATGATCGTTTCTGACATCGAAGCTAACGCCCTCTTAGAGAGCGTCACTAAGTTCCAC‑TGCGGGGTTATCTACGACTACTCCACCGCTGAGTACGTAAGCTACCGTCCGAGTGACTTCGGTGCGTATCTGGATGCGCTGGAAGCCGAGGTTGCACGAGGCGGTCTTATTGTGTTCCACAACGGTCACAAGTATGACGTTCCTGCATTGACCAAACTGGCAAAGTTGCAATTGAACCGAGAGTTCCACCTTCCTCGTGAGAACTGTATTGacactgtctc                            >  1:242713/11‑295 (MQ=255)`  
                                                                                                                                                                                                                                                                                  `aTAATG‑AATAGGAGAAATCAATATGATCGTTTCTGACATCGAAGCTAACGCCCTCTTAGAGAGCGTCACTAAGTTCCAC‑TGCGGGGTTATCTACGACTACTCCACCGCTGAGTACGTAAGCTACCGTCCGAGTGACTTCGGTGCGTATCTGGATGCGCTGGAAGCCGAGGTTGCACGAGGCGGTCTTATTGTGTTCCACAACGGTCACAAGTATGACGTTCCTGCATTGACCAAACTGGCAAAGTTGCAATTGAATCGAGAGTTCCACCTTCCTCGTGAGAACTGTATTGACACCCTtgtg                           <  1:298795/301‑1 (MQ=255)`  
                                                                                                                                                                                                                                                                                    `actcactaaaggagacacaccatGATCGTTTCTGACATCGAAGCTAACGCCCTCTTAGAGAGCGTCACTAAGTTCCAC‑TGCGGGGTTATCTACGACTACTCCACCGCTGAGTACGTAAGCTACCGTCCGAGTGACTTCGGTGCGTATCTGGATGCGCTGGAAGCCGAGGTTGCACGAGGCGGTCTTATTGTGTTCCACAACGGTCACAAGTATGACGTTCCTGCATTGACCAAACTGGCAAAGTTGCAATTGAACCGAGAGTTCCACCTTCCTCGTGAGAACTGTATTGACACCCTTgtgt                          <  1:65219/280‑1 (MQ=255)`  
                                                                                                                                                                                                                                                                                    `aaTC‑AATAGGATAAATCAATATGATCGTTTCTGACATCGAAGCTAACGCCCTCTTAGAGAGCGTCACTAAGTTCCAC‑TGCGGGGTTATCTACGACTACTCCACCGCTGAGTACGTAAGCTACCGTCCGAGTTACTTCGGTGCGTATCTGGATGCGCTGGAAGCCGAGGTTGCACGAGGCGGTCTTATTGTGTTCCACAACGGTCACAAGTATGACGTTCCTGCATTGACCAAACTGGCACAGTTGCAATTGAACCGAGAGTTCCACCTTCCTCGTGAGAACTGTATTGACACCCTTGtgtt                         >  1:34938/1‑301 (MQ=255)`  
                                                                                                                                                                                                                                                                                    `aaTC‑AATAGGATAAATCAATATGATCGTTTCTGACATCGAAGCTAACGCCCTCTTAGAGAGCGTCACTAAGTTCCAC‑TGCGGGGTTATCTACGACTACTCCACCGCTGAGTACGTAAGCTACCGTCCGAGTGACTTCGGTGCGTATCTGGATGCGCTGGAAGCCGAGGTTGCACGAGGCGGTCTTATTGTGTTCCACAACGGTCACAAGTATGACGTTCCTGCATTGACCAAACTGGCAAAGTTGCAATTGAACCGAGAGTTCCACCTTCCTCGTGAGAACTGTATTGACACCCTTgtgtc                         >  1:177798/1‑300 (MQ=255)`  
                                                                                                                                                                                                                                                                                     `aTC‑AATAGGAGAAATCAATATGATCGTTTCTGACATCGAAGCTAACGCCCTCTTAGAGAGCGTCACTAAGTTCCAC‑TGCGGGGTTATCTACGACTACTCCACCGCTGAGTACGTAAGCTACCGTCCGAGTGACTTCGGTGCGTATCTGGATGCGCTGGAAGCCGAGGTTGCACGAGGCGGTCTTATTGTGTTCCACAACTGTCACAAGTATGACGTTCCTGCATTGACCAAACTGGCAAAGTTGCAATTGAACCGAGAGTTCCACCTTCCTCGTGAGAACTGTATTGACACCCTTGtgttg                        >  1:8859/1‑301 (MQ=255)`  
                                                                                                                                                                                                                                                                                       `c‑AATAGGAGAAATCAATATGATCGTTTCTGACATCGAAGCTAACGCCCTCTTAGAGAGCGTCACTAAGTTCCAC‑TGCGGGGTTATCTACGACTACTCCACCGCTGAGTACGTAAGCTACCGTCCGAGTGACTTCGGTGCGTATCTGGATGCGCTGGAAGCCGAGGTTGCACGAGGCGGTCTTATTGTGTTCCACAACGGTCACAAGTATGACGTTCCTGCATTGACCAAACTGGCAAAGTTGCAATTGAACCGAAAGTTCCACCTTCCTCGTGAGAACTGTATTGACACCCCtgtctctta                      >  1:318452/1‑295 (MQ=255)`  
                                                                                                                                                                                                                                                                                            `aaggagacacaccatGATCGTTTCTGACATCGAAGCTAACGCCCTCTTAGAGAGCGTCACTAAGTTCCAT‑TGCGGGGTTATCTACGACTACTCCACCGCTGAGTACGTAAGCTACCGTCCGAGTGACTTCGGTGCGTATCTGGATGCGCTGGAAGCCGAGGTTGCACGAGGCGGTCTTATTGTGTTCCACAACGGTCACAAGTATGACGTTCCTGCATTGACCAAACTGGCAAAGTTGCAATTGAACCGAGAGTTCCACCTTCCTCGTGAGAACTGTATTGACACCCTTGTGTTGTCACGt                  >  1:46994/14‑301 (MQ=255)`  
                                                                                                                                                                                                                                                                                                `gaAATCAATATGATCGTTTCTGACATCGAAGCTAACGCCCTCTTAGAGAGCGTCACTAAGTTCCAC‑TGCGGGGTTATCTACGACTACTCCACCGCTGAGTACGTAAGCTACCGTCCGAGTGACTTCGGTGCGTATCTGGATTCGCTGGAAGCCGAGGTTGCACGAGGCGGTCTTATTGTGTTCCACAACGGTCACAAGTATGACGTTCCTGCATTGACCAAACTGTCAAAGTTGCAATTGAACCTAGATTTACACCTTCCTCGTAAGACCTGTATTAAAACTCTTGCTTTGTAACGTTTgt              >  1:179806/1‑300 (MQ=255)`  
                                                                                                                                                                                                                                                                                                   `aTCAATATGATCGTTTCTGACATCGAAGCCAACGCCCTCTTAGAGAGCGTCACTAAGTTCCAC‑TGCGGGGTTATCTACGACTACTCCACCGCTGAGTACGTAAGCTACCGTCCGAGTGACTTCGGTGCGTATCTGGATGCGCTGGAAGCCGAGGTTGCACGAGGCGGTCTTATTGTGTTCAACAACGGTCACAAGTATGACGTTCCTGCATTGACCAAACTGGCAAAGTTGCAATTGAACCGAGAGTTCCACCTTCCTCGTGAGAACTGTATTGACACCCTTGTGTTGTCACGTTTGattc           >  1:240877/1‑301 (MQ=255)`  

                                                                                                                                                                                                                                                                                                            `|`                                                                                                                                                                                                                                                                                                               
`GACAGACTGGCTGGAAGAAACCAGAAGTTACCAAGCTGAGTCATAAGGTGTTCGATAAGGACACTATGACCCACATCAAGGCTGGTGATTGGGTTAAGGTTGACATGGGAGTTGTTGGTGGATACGGCTACGTCCGCTCAGTTAGTGGCAAATATGCACAAGTGTCATACATCACAGGTGTTACTCCACGCGGTGCAATCGTTGCCGATAAGACCAACATGATTCACACAGGTTTCTTGACAGTTGTTTCATATGAAGAGATTGTTAAGTCACGATAATC‑AATAGGAGAAATCAATATGATCGTTTCTGACATCGAAGCTAACGCCCTCTTAGAGAGCGTCACTAAGTTCCAC‑TGCGGGGTTATCTACGACTACTCCACCGCTGAGTACGTAAGCTACCGTCCGAGTGACTTCGGTGCGTATCTGGATGCGCTGGAAGCCGAGGTTGCACGAGGCGGTCTTATTGTGTTCCACAACGGTCACAAGTATGACGTTCCTGCATTGACCAAACTGGCAAAGTTGCAATTGAACCGAGAGTTCCACCTTCCTCGTGAGAACTGTATTGACACCCTTGTGTTGTCACGTTTGATTCATTCCAACC  >  NC_001604.1/14057‑14656`

  

Alignment Legend

Aligned base mismatch/match (shaded by quality score): `ATCG/ATCG` `< 3 ≤` `ATCG/ATCG` `< 11 ≤` `ATCG/ATCG` `< 23 ≤` `ATCG/ATCG` `< 35 ≤` `ATCG/ATCG` `< 38 ≤` `ATCG/ATCG`

Unaligned base: `atcg`    Masked matching base: `atcg`    Alignment gap: `‑`    Deleted base: `‑`

Reads not counted as support for junction

read\_name Not counted due to insufficient overlap past the breakpoint.

read\_name Not counted due to not crossing MOB target site duplication.
